# A Ward Snowstorm at Bath

**Published:** 1914-01-03

**Authors:** 


### A Ward Snowstorm at Bath.

If anyone had doubted the existence of the spirit of Christmas in hospital life, a visit to the Royal United Hospital, Bath, must have set all doubts at rest. As one entered the Victoria Ward one was met by a "blinding snowstorm"—a beautiful and striking effect produced by hundreds of strings hanging from the ceiling with pieces of cotton-wool (non-inflammable) fixed at intervals down each string, which, together with more "snow" on window ledges, robins on twigs, and the addition of blue lamp-shades, gave quite a wintry appearance. In the Duncan Wards scarlet was the prevailing colour, which, with the addition of holly and evergreens and a multitude of "snow," could not fail to be recognised as "Christmassy," while much the same scheme was followed out in the Albert Ward; but here the colours were red and yellow. The Helena Ward was Japan—almond blossom, Japanese umbrellas, and kimonos—the latter worn by the patients—here held the field, while our old friend the Japanese lantern was clearly in evidence. The Cambridge Ward confined itself to the use of chrysanthemums, and a pretty effect was secured by the help of pink lamp-shades. But, of course, the centre of attraction was, as usual, the Prince of Wales's Ward (children), and no trouble was spared to make it really beautiful. Working from the centre gas pendant, the staff converted the ward into a huge maypole. Streamers of all hues hung from the centre to each cot, which was itself decorated, while the occupants were arrayed in coloured garments in keeping with the streamer attached to their bed. A mighty Christmas-tree occupied one end of the ward, and smiling faces were the order of the day.

